# Ultrasound-Assisted alkaline hydrolysis of feather keratin using a cup horn sonoreactor

**DOI:** 10.1016/j.ultsonch.2025.107733

**Published:** 2025-12-28

**Authors:** Nidal Del Valle Raydan, Antoine Loquet, Birgit Habenstein, Brice Kauffmann, Gregory Chatel, Eduardo Robles

**Affiliations:** a University of Pau and the Adour Region, E2S UPPA, CNRS, IPREM-UMR 5254, Mont de Marsan, France; b Univ. Bordeaux, CNRS, Bordeaux INP, CBMN, UMR 5248, IECB, F-33600 Pessac, France; c Univ. Bordeaux, CNRS, INSERM, IECB, US1, UAR 3033, F-33600 Pessac, France; d Université de Savoie Mont Blanc, CNRS, EDYTEM, 73000 Chambéry, France

**Keywords:** Feathers, Keratin, Sonochemistry, Cuphorn, Temperature effect

## Abstract

This study explores the potential of ultrasound-assisted alkaline hydrolysis, employing a cup horn sonoreactor, for the sustainable extraction of keratin from duck feather waste. Unprecedented in its approach, this research evaluates the system’s efficacy in maintaining the structural integrity of cystine—a crucial amino acid—through controlled hydrolysis processes, or promoting disulfide bond rupture and regeneration upon precipitation. By using the unique advantages of the cup horn system, including homogeneous energy distribution and gentle processing, this investigation aims to overcome the limitations of hydrothermal treatments. The obtained keratins were analyzed using advanced spectroscopic, microscopic, and thermal analysis techniques (ATR-IR, Raman, SDS-PAGE, SEM, 13C CP-MAS NMR, XRD, and TGA). These analyses allowed the unveiling of the reaction pathways and structural changes in keratin under various temperatures in alkaline conditions. Lower temperatures (35 °C) favored the preservation of native disulfide linkages, while higher temperatures (75 °C) enhanced disulfide bond rupture and reformation. An intermediate temperature (55–65 °C) offered a balance between structural integrity and yield. This innovative method represents a significant advancement in feather waste valorization, providing a scalable and adaptable platform to tailor keratin properties such as yield, thermal stability, or disulfide bond regeneration, according to specific application needs.

## Graphical abstract

**Figure:**
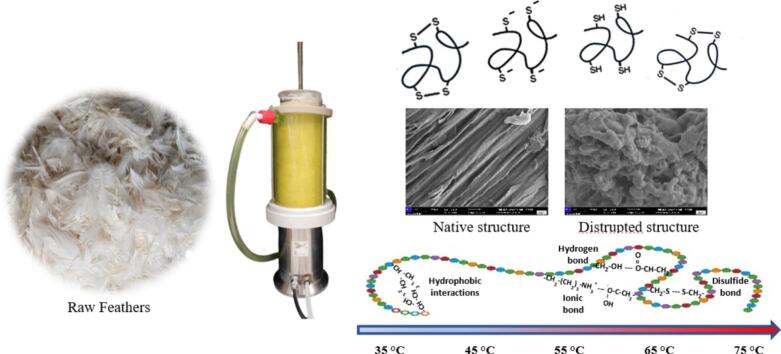

## 1 Introduction

Keratinous materials, encompassing feathers, wool, horns, and hooves, are recognized as valuable waste products, rich in protein and sulfur, predominantly marked by a high cystine content of 7–13 % [1]. This differentiates keratins from other structural proteins, such as collagen and elastin. Feather keratin's robust and resistant nature, stemming from its dense disulfide cross-linkages, hydrogen and ionic bonds, and hydrophobic interactions, poses a significant challenge for large-scale utilization and valorization[2]. The sustainable disposal of feather waste poses a significant challenge, especially within the poultry industry. This issue is further complicated by the unique molecular composition of feathers, characterized by the presence of β-keratin with pleated sheets known as corneous beta-proteins [3], [4]. These structures are remarkably stabilized by various bonds, where cystine's disulfide bonds notably contribute to the robust nature of keratin, thereby resisting dissolution in organic solvents [5].

Numerous research studies have shown potential for repurposing feather waste, but as of October 2023, five million tons of feather waste are still landfilled annually, indicating a significant gap between research potential and industrial application [6]. However, the valorization of keratinous residues is correlated with hydrolysis steps. Usually, the feather hydrolysis method consists of a hydrothermal process at high pressures (from 300 to 350 kPa) and high temperatures (from 133 to 150 °C) [7]. However, traditional hydrothermal treatments for feather hydrolysis, while effective in breaking down keratin structures, are inefficient in yield, time-intensive, and lead to the deterioration of amino acid quality through racemization and oxidation [8]. In contrast, as an eco-friendly and non-thermal alternative, ultrasound technology promises to transform keratin hydrolysis by leveraging cavitation effects to alter protein hydration, size, hydrophobicity, and conformation, thus enhancing protein solubility and hydrolysis [9], [10].

Alkaline hydrolysis, while extensively studied, has not been thoroughly explored in combination with ultrasound. Given cystine's structural and nutritional importance, its preservation during protein extraction is crucial. However, cystine's susceptibility to rapid decomposition in alkaline conditions, especially when coupled with heat, poses a significant challenge [1]. The formation of lanthionine, a byproduct of cystine degradation that lacks nutritional value, is undesirable in a process complicated by the temperature-dependent stability of keratin's cystine and its compactness provided by its disulfide bonds [11], [12].

**Fig. 1:**
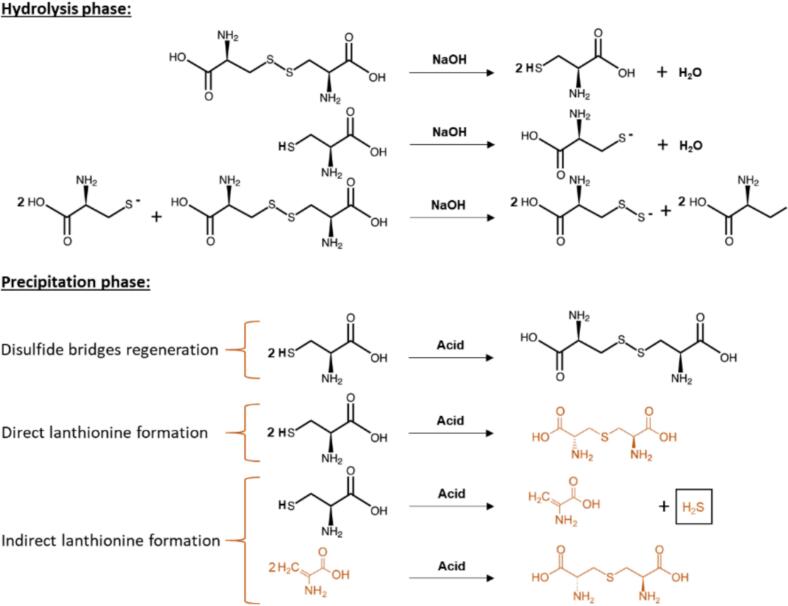
Reaction pathways during the ultrasound-assisted alkaline hydrolysis of feathers at elevated temperatures and subsequent acidification to pH 4.5 for keratin precipitation,

The innovative use of ultrasound technology, specifically through a cup horn sonoreactor, offers a new dimension to keratin regeneration from feather waste. The cup horn system has not been previously applied for ultrasound-assisted alkaline hydrolysis of feathers, presenting an opportunity to explore its efficacy. Compared with conventional ultrasonic probes, the cup horn system provides a uniform distribution of ultrasound energy, facilitating a consistent and precisely controlled hydrolysis process. Furthermore, its gentle processing feature reduces the risk of overheating or damaging sensitive keratin structures, making it an effective and sustainable way to manage feather waste [13]. The advantage of ultrasound-assisted processes, particularly with a cup horn system, lies in the ability to facilitate hydrolysis at lower temperatures and shorter times, thereby preserving the structural integrity of valuable amino acids like cystine. In alkaline hydrolysis, NaOH is a potent agent that can disrupt the non-covalent bonds at lower temperatures and shorter times. In comparison, with longer times and higher temperatures, it assists in breaking and potentially reforming the disulfide bridges in keratin upon precipitation with acid, as depicted in the provided image of pathways in Fig. 1. This delicate interplay between NaOH, time, and temperature is crucial for maintaining the cystine content, as alkaline conditions can rapidly decompose cystine, resulting in undesirable byproducts such as lanthionine and H_2_S.

adapted from [12], [14], [15], [16], [17], [18].

The outcomes of these reactions depend heavily on the processing parameters, such as temperature and the duration of exposure to alkaline conditions. The study's comprehensive characterization of the regenerated keratin hydrolysates will employ techniques such as Attenuated Total Reflectance Infrared (ATR-IR) spectroscopy, Raman spectroscopy, SDS-PAGE, X-ray Diffraction (XRD), Nuclear Magnetic Resonance (NMR), Scanning Electron Microscopy (SEM), Thermogravimetric Analysis (TGA), and Differential Scanning Calorimetry (DSC), to elucidate the mechanisms at play at each temperature setting. These techniques will help understand how the different bonds within keratin are affected during hydrolysis and the subsequent impact on the keratin's properties. This research aims to chart new territory in the sustainable and efficient processing by investigating the ultrasound-assisted alkaline hydrolysis mechanisms at varying temperatures in a cup horn sonoreactor. The objective is to develop a process that is not only environmentally friendly but also preserves the valuable components of keratin, making it a viable resource for creating high-quality materials.

## 2 Materials and methods

### 2.1 Materials

The raw feather (RF) material utilized was white feathers from the Mullard duck, generously supplied by an industrial partner (Plum'Export, Saint-Sever, France). The sample corresponds to the fraction of feathers discarded after selecting down and bristles, which the company sells for quilts and down jackets. Random feathers were analyzed in a prior study, having a moisture content of 9.15 %, a protein content of 82.97 %, a fat content of 0.98 %, and an ash content of 0.68 % [16]. Sodium hydroxide (NaOH, CAS number 1310–73-2), citric acid (C.A., HOC(CO_2_H) (CH_2_CO_2_H)_2_, 77–92-9) used in this research work were purchased from Sigma-Aldrich.

### 2.2 Methods

#### 2.2.1 Ultrasound-assisted alkaline hydrolysis of raw duck feather

The cleaned and ground feathers (to 5 mm) were combined with a 3 % wt NaOH solution at a 10:100 ratio. This mixture was processed using a 22 kHz Lab500 homogenizer within a cup horn reactor (SiLabTec, France, 119 mm diameter, 150 W maximum power). Hydrolysis was conducted under continuous ultrasonic irradiation at 80 % amplitude across different temperatures ranging from 35 °C to 75 °C, while mechanically stirring at 500 rpm in a 500 mL jacketed vessel until a homogeneous colloid was achieved.

##### 2.2.1.1 Keratin regeneration

The keratin colloid was filtered to remove the insoluble feather parts, then left to cool and precipitated to pH 4.5 with citric acid, followed by dialysis. The dialysis was carried out over three days using a 6–8 kDa molecularporous membrane tubing (Spectra/Por®1, Spectrum Labs, USA). Neutralized samples were dried with a lyophilizer (Alpha 1–4, Martin Christ GmbH, Osterode am Harz, Germany).

##### 2.2.1.2 Data acquisition

The collected dataset measured included the hydrolysis time (t, in minutes), the energy density (Ed, in J/m3), and the absolute yield (AY, in %) at every temperature upon obtaining a homogeneous colloid. The nominal energy density (Ed) was calculated based on the electrical energy consumption (E, in kilowatt-hours), as recorded by the Cup Horn sonoreactor's control unit, and normalized to the reaction volume using Eq. 1.

Ed (J/m^3^) = E (kWh)* 3.6*10^6^/V (m^3^) (1)

Where:

E is the recorded electrical energy consumed during one hydrolysis time (in kWh), 3.6 × 10⁶ converts kilowatt-hours into joules (1 kWh = 3.6 million J), and V is the reaction volume, fixed at 0.00045 m^3^ (450 mL).

This nominal Ed reflects the electrical input energy to the sonoreactor, not the acoustic energy directly delivered to the reaction medium. While calorimetric or hydrophone-based calibrations would provide more precise values of actual acoustic energy transfer, the use of nominal energy density allows for practical, reproducible comparisons between different ultrasonic configurations (e.g., Cup Horn vs. probe-type) under clearly defined volume and power input conditions.

The absolute yield (AY) of the keratin hydrolysate (KH) obtained at each temperature, denoted by Eq. 2, was calculated relative to the initial keratin content of RF (0.83 × M_0_), rather than to the total feather mass (M_0_). The keratin content of RF was determined through Kjeldahl's method (82.97 ± 0.98 %)[19].

AY (%) = [M_dry_/ 0.83 M_0_]*100 (2)

Where:

M_0_ is the initial mass of raw feathers used in hydrolysis, M_dry_ is the mass of lyophilized recovered keratin, and 0.83 M_0_ is the estimated initial keratin fraction in RF, obtained from Kjeldahl nitrogen determination.

## 3 Characterization methods

### 3.1 Colorimetric analysis

The color of keratin colloids obtained after ultrasound-assisted alkaline hydrolysis and the dry keratin post-lyophilization across temperatures ranging from 35 °C to 75 °C was measured using an X-rite Color Ci62S colorimeter (X-Rite Inc., MI, USA), which was pre-calibrated with white and black standards. The CIE color scale was chosen to obtain the color values, which were expressed as L* (lightness), a* (redness), and b* (yellowness) values.

### 3.2 Chemical and structural characterization

SDS-PAGE analysis was performed using Laemmli's method[20] to analyze RF and the KH obtained at different temperatures. A bovine albumin standard (2 mg/mL) was used as a reference. All samples were incubated for five minutes at 95 °C in 2x buffer of 250 mM Tris-HCl (pH 6.8), 8 % SDS, 40 % glycerol, 8 % beta-mercaptoethanol, and 0.02 % bromophenol blue. For the gel, 10 μL of Prestained Protein Ladder (3.5–260 kDa) was loaded in lane 1, while 10 µL of the different samples and bovine albumin were loaded in separate wells of the subsequent lanes. The electrophoresis process utilized Bolt™ 4–12 % Bis-Tris Plus Gel (Invitrogen™ NW04120BOX, USA) and MES SDS running buffer. The gels were run at 180 V until the dye front reached the bottom of the gel. Following electrophoresis, the gels were stained with Coomassie Brilliant blue R 250 and rinsed with 5 % acetic acid and 20 % methanol until a clear background was observed.

Functional groups of the feathers and the KHs were analyzed using Attenuated Total Reflectance (ATR) mode in an ATR-IR spectrometer (FT/IR-4700, Jasco). All spectra were recorded over a 4000–400 cm^−1^ range using 64 scans and 2 cm^−1^ resolution. These spectra were normalized to the amide I band at 1632 cm^−1^.

RAM II module was employed to obtain the Raman spectra in the spectral range between 800 and 3600 cm^−1^ using a diode-pumped Nd:YAG laser with an excitation wavelength of 1064 nm at a laser power of 150 mW. Each spectrum had an average of 250 scans.

Solid-state NMR experiments were recorded at room temperature on a 5500 MHz spectrometer (Bruker BioSpin) using a 4 mm double resonance probe under magic-angle spinning conditions (11 kHz). ^13^C-detected spectra were obtained using an initial cross-polarization with a contact time of 1 ms or an INEPT polarization transfer. High-power decoupling (SPINAL-64) was applied during acquisition. Chemical shifts were calibrated and referenced with DSS.

X-ray diffraction experiments were performed on a Rigaku FRX rotating anode at the copper kα wavelength using a Hybrid Pixel detector (HyPix6000 from Rigaku). MicroMeshes from MiTeGen® were used as powder sample holders on the goniometer head. Each diffraction pattern represents a 360° rotation along the φ axis, with an exposure time of 720 s. No corrections, such as a smooth filter or baseline correction, were applied to the data. WinPLOTR (https://cdifx.univ-rennes1.fr/winplotr/winplotr.htm) was used to generate the 2D plots.

Morphology was studied using scanning electron microscopy (SEM). The samples were mounted onto specific stubs and coated with platinum using a sputter coater (Q150T, Quorum Technologies, Kent, UK) to determine the particle morphology. Observations are conducted at 2 kV, in a high vacuum mode, with a Gemini SEM 300 FESEM (Zeiss, Oberkochen, Germany).

### 3.3 Thermal analysis

The thermal resistance of the different ground feathers and KH was measured using a TA Q500 thermogravimetric analysis; samples were heated from 30 °C to 800 °C at a rate of 10 °C/min under a nitrogen atmosphere. The melting temperatures of different ground feathers and KH were measured using a TA Q20 differential scanning calorimetry under a continuous nitrogen purge at a heating rate of 10 °C/min over a temperature range of −50 °C °C to 250 °C.

## 4 Results and Discussion

### 4.1 Analysis of Yield, energy Density, and reaction time as a Function of temperature

**Fig. 2:**
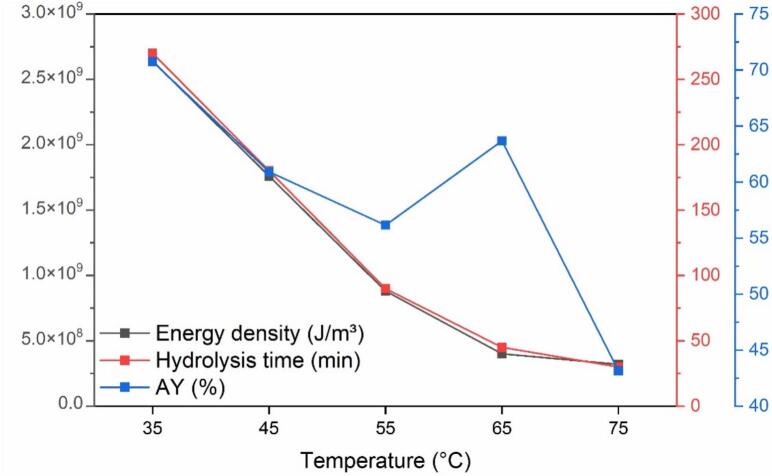
Correlation of Energy Density, Hydrolysis Time, and Absolute Yield of KH in the Ultrasound-Assisted Alkaline Hydrolysis of RF at Various Temperatures.

In Fig. 2, the impact of temperature on the ultrasound-assisted alkaline hydrolysis of duck feather keratin is presented, focusing on energy density (J/m^3^), hydrolysis time (minutes), and absolute yield (AY, %) across a temperature range from 35 °C to 75 °C. As the temperature increased from 35 °C to 75 °C, energy density and hydrolysis time initially showed a piecewise linear correlation, which suggests a different linear relationship within separate temperature intervals: one from 35 °C to 55 °C and another from 65 °C to 75 °C. The inflection point at 55 °C demonstrates a shift in the hydrolysis reaction dynamics, with an enhanced hydrolysis efficiency observed after this point. On the other hand, the absolute yield is maximum at the lowest temperature of 35 °C (73 %) and then decreases with rising temperatures, except for a pronounced peak at 65 °C (64 %). This peak at 65 °C may resemble the performance of direct probe-type ultrasound hydrolysis at 75 °C, which also achieved an AY of approximately 72 % under similar alkaline conditions and energy densities [21]. Both values significantly exceed the yield from a conventional hydrothermal alkaline process, which is about 23 % [21]. Compared to Sommer et al. (2024), which reached up to 72 % yield after 32 h using similar feather to NaOH ratios and high shaking speeds [22]. The Cup Horn approach achieves similar or higher yields in less time and with lower mechanical energy input, highlighting its potential as a rapid and energy-efficient method. This may suggest a temperature-specific response, likely the optimal regeneration of disulfide bridges, which are crucial for keratin’s structural stability. The diminishing yield at the highest temperature (75 °C) is likely attributed to the protein degrading into smaller, non-precipitable fragments.

### 4.2 Colorimetric analysis

**Fig. 3:**
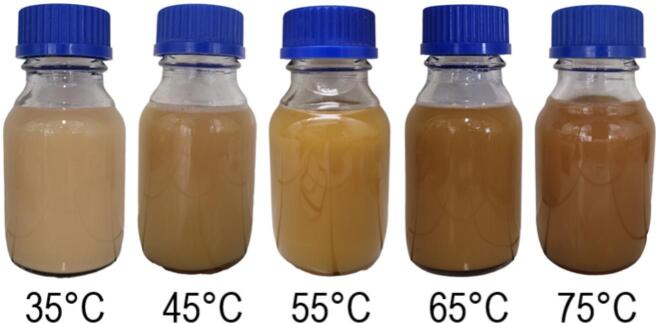
Keratin colloids from feather hydrolysis at temperatures from 35 °C to 75 °C show a temperature-dependent transition from opaque light to translucent dark colors.

Visual observation of keratin colloids after ultrasound-assisted alkaline hydrolysis across varying temperatures (35 °C to 75 °C) shows a progressive darkening with temperature increase (Fig. 3). This darkening is likely a result of multiple chemical reactions affecting the protein structure. At lower temperatures, a predominance of hydrogen bonds and hydrophobic interaction disruptions is possible, with disulfide bonds remaining almost intact. This results in lighter, more opaque colloids due to light scattering by larger or irregularly shaped protein fragments. As the temperature rises, the hydrolysis intensifies to include disulfide bond cleavage, yielding less opaque colloids. These changes suggest a reduction in protein fragment size or a more extensive breakdown into soluble forms, which minimally scatter light.

**Fig. 4:**
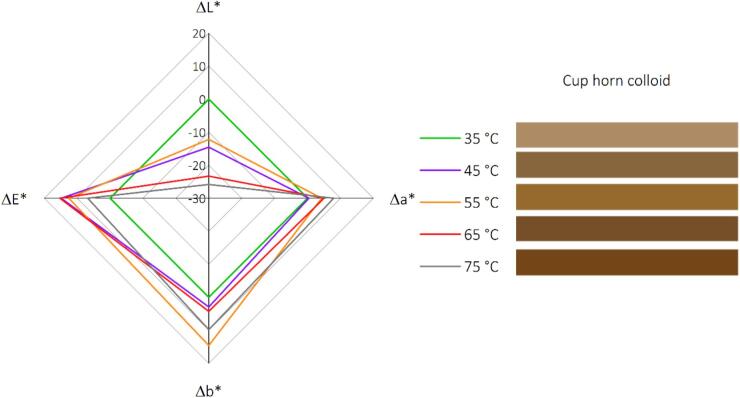
The colorimetric data of keratin colloids resulting from ultrasound-assisted alkaline hydrolysis at various temperatures.

More insights regarding the color changes are available in Fig. 4, which illustrates the colorimetric response of keratin colloids post-ultrasound-assisted alkaline hydrolysis across varying temperatures (35 °C to 75 °C). A notable trend in the L* values is observed, with a consistent decrease from 60.16 at 35 °C to 34.32 at 75 °C. This diminishing luminance with increasing temperature confirms the darkening of the sample, which indicates thermal effects on the sample's composition or structure that result in less light reflection. In parallel, a* values increase from 7.79 to 15.74 across the temperature range, indicating a shift toward redder tones. Similarly, the b* values increase from 25.21 to 35.02, pointing to a shift toward yellower tones. These shifts in a* and b* values suggest that as the temperature rises, the sample's color moves toward the warmer end of the spectrum, possibly due to thermochromic behavior or a change in the concentration of chromophores. The ΔE* values, representing the color differences relative to the previous temperature condition, provide insight into the perceptual change in color. The ΔE* values range from 14.83 between 35 °C and 45 °C to 6.78 between 65 °C and 75 °C. The highest ΔE* value is 15.24, occurring between 55 °C and 65 °C, reflecting the most significant perceptual change over the temperature intervals studied. It is essential to note that while ΔE* values above 1.0 are typically noticeable to the human eye, those above 6.0 are considered significant color shifts. In this context, all measured ΔE* values suggest perceptible changes, with some reaching a substantial color change, underscoring the keratin's sensitivity to temperature.

**Fig. 5:**
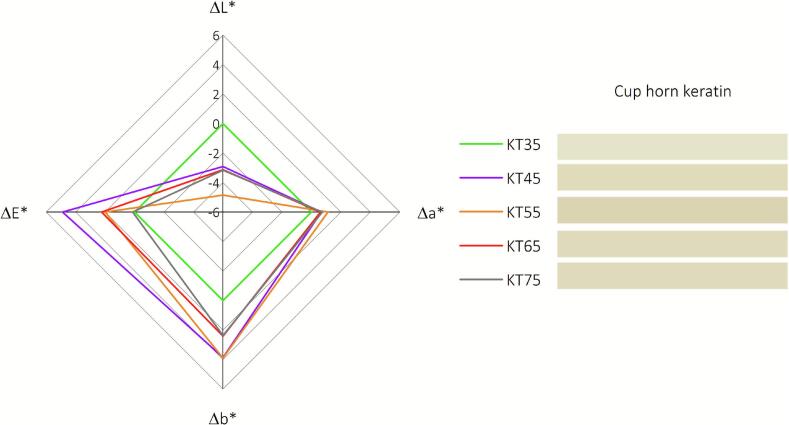
The colorimetric data of recovered keratin resulting from ultrasound-assisted alkaline hydrolysis at various temperatures. Chemical and structural characterization.

The colorimetric data presented in Fig. 5 provide quantitative insights into the optical properties of recovered dry keratin resulting from ultrasound-assisted alkaline hydrolysis at various temperatures, ranging from 35 °C to 75 °C. A notable descending trend in the L* values is observed from 89.84 at 35 °C to 86.68 at 75 °C, suggesting a progressive darkening of the keratin as the temperature rises, with keratin recovered at 55 °C showing the highest darkening. This darkening effect could be due to the formation of colored compounds or alterations in the keratin's structure, which aligns with the cleavage and possible rearrangement of disulfide bridges. The a* values indicate a transition from a slight greenish hue at 35 °C (−0.36) towards a reddish tone at 75 °C (0.38), with the keratin processed at 55 °C having the highest a* value (0.76). In parallel, the b* values increase from 13.09 at 35 °C to 15.40 at 75 °C, shifting from a blue to a yellow tone, with keratin recovered at 55 °C registering the highest b* value. The darkening and color shifts noted in the keratin samples, particularly at 55 °C, may be due to the temperature's effect on disulfide bond integrity, resulting in structural changes and the exposure or breakdown of chromophoric elements. This aligns with the observed inflection at 55 °C in hydrolysis efficiency, confirming a notable transition in reaction dynamics at this temperature. The (ΔE*) values indicate minimal color differences across all temperatures, with the slightest change noted between 65 °C and 75 °C (ΔE* = 0.13). The most notable shift occurs between 35 °C and 45 °C (ΔE* = 4.89), and a significant alteration is also seen between 55 °C and 65 °C (ΔE* = 2.24), highlighting the substantial visual impact of temperature on keratin coloration during these intervals, aligning with that of keratin colloids. Despite these variations, the overall pale-white appearance of keratin suggests that NaOH drives the browning effect through the unfolding and exposure of chromophoric groups. However, their SS bridge reconstitution at higher temperatures refolds these chromophoric groups, resulting in minimal differences across all obtained keratins.

**Fig. 6:**
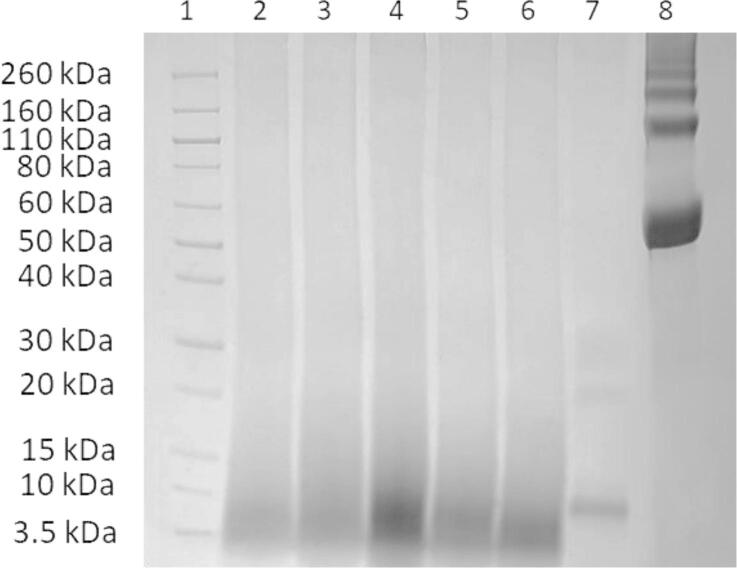
SDS-PAGE of feather and protein isolates: Lane (1) prestained protein Ladder (3.5–260 kDa), Lanes (2 to 6) are the recovered keratin from increasing hydrolysis temperature (35 °C to 75 °C), Lane (7) raw feather, and Lane (8) albumin standard.

The SDS-PAGE analysis presented in Fig. 6 reveals significant differences in the molecular weight profiles of keratin across a temperature gradient from 35 to 75 °C (Lanes 2 to 6). At lower temperatures (35 and 45 °C), the hydrolysis yields keratin fragments that exhibit clear and distinct bands below 10 kDa, suggesting partial cleavage of keratin structures. These bands indicate that the hydrolysis process has begun, but the extent of breakdown is limited, likely due to insufficient energy to overcome the stability of the keratin disulfide bridges. Remarkably, the sample treated at 55 °C displays a concentrated band with accompanying smearing, indicating a more extensive hydrolysis process. This pattern suggests that 55 °C may represent a starting temperature threshold for the cleavage of disulfide bridges within the keratin. The smearing observed could result from the hydrolysis conditions facilitating the formation of a diverse range of hydrolysis products, reflecting heterogeneity in the sample. As the temperature increases further to 65 and 75 °C, the smearing of the bands decreases compared to the 55 °C, yet remains more concentrated than at lower temperatures. This observation suggests that while higher temperatures promote keratin breakdown into smaller peptides, there is a shift towards a more homogeneous distribution of molecular weights.

In contrast, Lane 7, representing the reference feather (RF), displays a distinct keratin band with a molecular weight of approximately 10 kDa. This observation aligns with Woodin's research [23], suggesting that feather keratin predominantly consists principally of β keratin having molecular weights ranging between 10 –14 kDa [24]. Additionally, two notable protein bands were identified: the first between 20 and 25 kDa, and a less distinct second band between 30 and 40 kDa. These can be attributed to less intense α keratin of higher molecular weights [25].

**Fig. 7:**
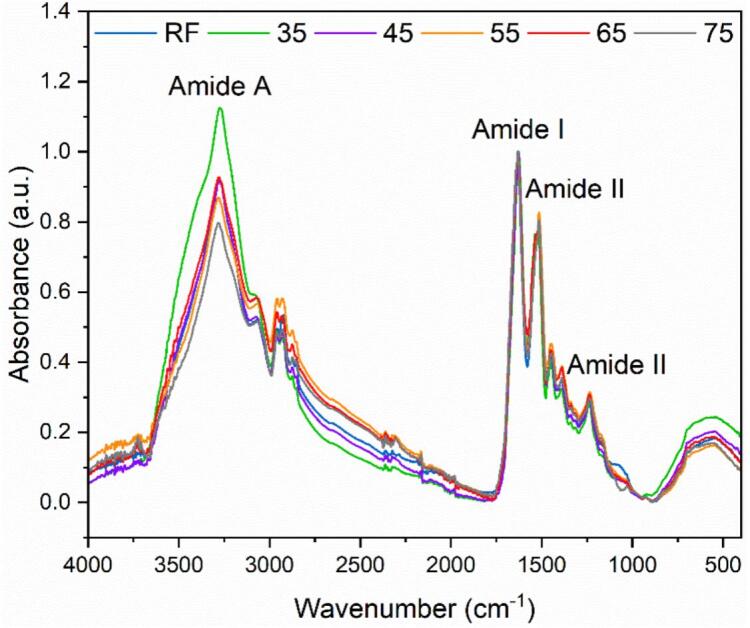
FTIR spectra showing the absorbance profiles of raw feather and recovered keratin from increasing hydrolysis temperature (35 °C to 75 °C).

ATR-FTIR analysis of keratin hydrolysates at different temperatures compared with Raw Feather (RF) exhibits the characteristic absorption bands of proteins (Fig. 7). The amide A band from 3500 cm^−1^ to 3200 cm^−1^ is attributed to stretching vibrations of −O-H (bonded water) and −N-H. The broad absorption band region from 3500 cm^−1^ to 3200 cm^−1^ (amide A) indicates α-helix structure [26] and hydrogen bond strength (amide A). The data suggest that keratin treated at lower temperatures maintains stronger hydrogen bonds, indicative of a more intact α-helix structure. However, the results at 65 °C indicate a deviation from this trend, which may point to this temperature facilitating an optimal balance between disruption and regeneration of disulfide bridges, where the re-establishment of α-helix structure and hydrogen bonds is also prominent. The asymmetric and symmetric C-H stretching (CH_3_) reflect at 2960 and 2925 cm^−1,^ respectively [27]. The increase in the symmetric stretching of C-H bonds in methylene groups (–CH_2_-), at 2850 cm^−1^ across all keratin hydrolysates in contrast to the RF, suggests alterations in the keratin structure, leading to a transformation that may involve the formation of other amino acids with alkyl side chains. The amide I band (1700–1600 cm^−1)^ is mainly due to the CO stretching vibration and is directly related to the protein backbone conformation. The amide II band (1580––1480 cm^−1^) is attributed to the NH bending vibration and CN stretching vibration and is predominantly known for its sensitivity to the protonation state of the peptide unit. Finally, the amide III band (1300–1220 cm^−1^) results in phase combination of CN stretching and NH in plane bending and depends on the nature of side chains. These amide bands, especially amide I, are crucial for determining the protein's secondary structure [28].

**Fig. 8:**
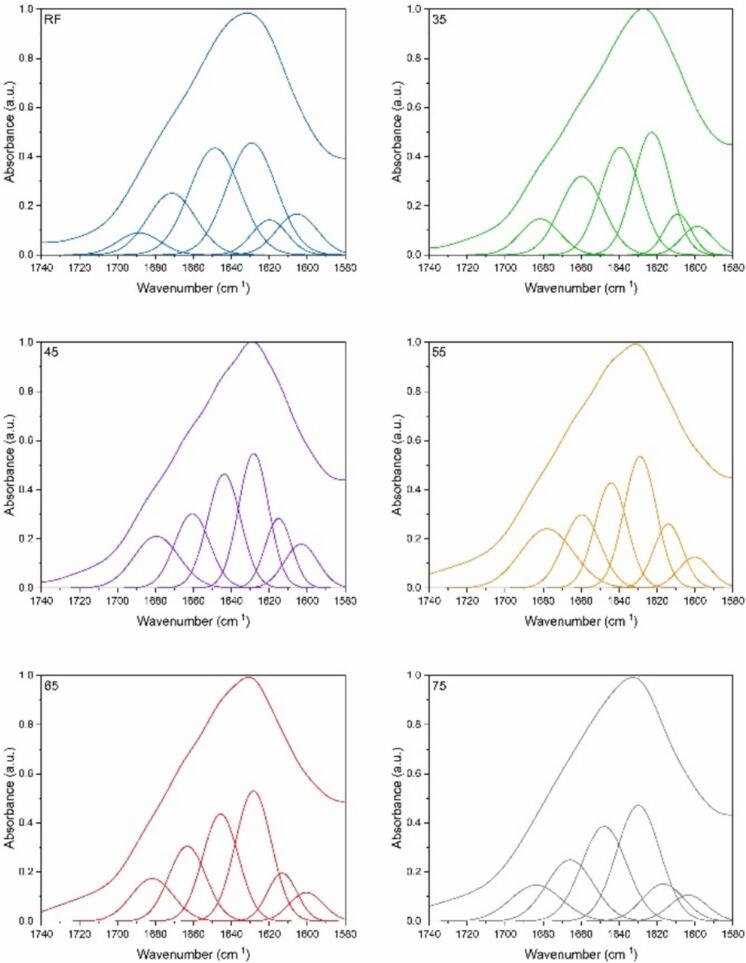
Curve fitting of the amide I band for raw feather and recovered keratin from increasing hydrolysis temperature (35 °C to 75 °C).

Analysis of the Amide I band through Fourier deconvolution, second derivative, curve fitting, and spectral subtraction reveals the presence and proportion of α-helices, β-sheets (intermolecular antiparallel (inter. AP); intramolecular Parallel (intra. P); and turn/ random coil structures, β-sheets (inter AP)/ side chains in Fig. 8 and Table 1. In general, α-helical structures have a band at wavenumbers 1650–1658 cm^−1^; β-sheet structures tend to have bands between 1620 and 1640 cm^−1^ and between 1670 and 1695 cm^−1^; random coil structures occur at around 1644 cm^−1^ [28]. The peak fitting analysis indicates a pronounced presence of aggregate strands at 45 °C, implying a key temperature for initiating unfolding and aggregation, leading to the exposure and interaction of hydrophobic regions within the keratin. This is accompanied by a reduction in both β-sheet (intra. P) and α-helix structures, lacking substantial disulfide bond regeneration, in contrast to their higher occurrence at 55 °C. The diminished aggregation at 55 °C may reflect disaggregation due to bond cleavage and reformation. At 45 °C, a pronounced intermolecular antiparallel β-sheet presence likely signifies new β-sheet formation.

**Table 1:** The secondary structure composition of raw feather and recovered keratin was recovered from increasing hydrolysis temperature (35 °C to 75 °C).

Peak	Assignment	RF		35		45		55		65		75	
		Position	%	Position	%	Position	%	Position	%	Position	%	Position	%
I	Aggregate strands	1605.6	9.2	1599	5.6	1603.3	9	1600.4	6.1	1600.7	6.2	1603.7	5.9
II	β-sheet (inter. AP)	1619.9	6.9	1609.1	6.9	1615.1	11.4	1614.1	11.4	1613.2	9.1	1616.7	9.4
III	β-sheet (intra. P)	1629.3	30.9	1622.8	28.3	1628.2	24.9	1628.9	26	1628.3	30.2	1629.8	30.4
IV	α-helix	1648.7	16.4	1639.3	27.1	1643.8	23.2	1644.2	20.8	1645.6	24.7	1647.9	25.8
V	β-turn/random coil	1671.4	5.2	1659.7	21.4	1660.6	16	1659.6	16.6	1663.2	17.9	1665.8	17.4
VI	β-sheet (inter AP)/side chains	1688.6	1.3	1681.5	8.9	1679.4	14.4	1678	19.2	1681.6	11.5	1683.7	11.2

On the other hand, the considerable β-sheet content in keratin obtained at 35 °C suggests that under mild conditions, the native β-structure of feathers is largely maintained, with partial protein unfolding exposing side chains within β-sheet (inter AP)/side chains: 6.9) and (β-sheet (intra. P), whereas α-helices were increased to a maximum at this temperature, which is correlated with the results of amide A in the ATR-IR spectrum. β-turns and random coils are consistently more prevalent in all keratin samples than the original feather material, with minimal variance across the 45 to 75 °C. This constancy underscores their potential as stable intermediates in the hydrolysis-induced unfolding and refolding pathways of keratin.

**Fig. 9:**
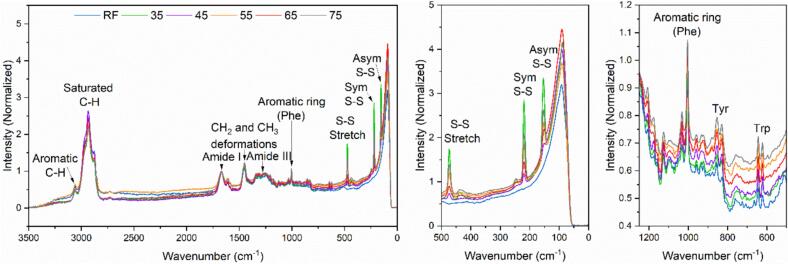
Raman spectroscopy shows the absorbance profiles of recovered keratin from increasing hydrolysis temperature (35 °C to 75 °C). The raw feather (RF) is shown for comparison.

Raman spectroscopy has been highly influential in studying protein conformational changes, particularly in analyzing structural and functional shifts up to protein unfolding or denaturation [29]. Raman spectra (Fig. 9) show the absorbance profiles of the keratin hydrolysates recovered from increasing hydrolysis temperature (35 °C to 75 °C), along with the raw feather (RF). The most outstanding peak, centered near 1670 cm^−1^, was used to normalize all samples. This peak has been assigned to the amide I vibration mode, mainly involving the extension of C=O and C–N. The amide III peak (≈1204 cm^−1^, ≈1250 cm^−1^, ≈1278 cm^−1^, ≈1311 cm^−1^, and ≈1343 cm^−1^) mainly involves the extension of C–N and the planar transition of NH [30].

Specific to the thiol-containing amino acid cysteine, the S-H stretching vibrations are identified at 2552 cm^−1^, 940 cm^−1^, 870 cm^−1^, 822 cm^−1^, 774 cm^−1^, and 537 cm^−1^. In contrast, the peak at ≈500 cm^−1^ is characteristic of cystine's disulfide bond (S-S). Within the lower frequency region of the spectrum, the peaks at ≈150 cm^−1^ and ≈220 cm^−1^ represent asymmetric S-S bending and symmetric S-S bending, respectively [31]. The peak at 473 cm^−1^, associated with a shoulder at ≈467 cm^−1^, deviates from the typical S-S stretch in cystine around ≈500 cm^−1^. The shoulder feature is potentially attributable to metal thiolates or the thioether structures characteristic of lanthionine formations [32]. The observed spectral features suggest the cleavage and reformation of cystine disulfide bonds and the possible creation of other sulfur-containing entities, such as lanthionines or thiolates. However, the complexities of these molecular transformations cannot be entirely ascertained through Raman spectroscopy alone. It is important to note that lanthionine typically forms at elevated temperatures, but under the unique conditions of alkaline hydrolysis coupled with ultrasound over extended durations, the necessary conditions for its formation may occur.

The pronounced peaks at 153 cm^–1^, 220 cm^–1^, and 473 cm^–1^ in all keratin hydrolysates suggest significant alterations in the disulfide bonding patterns (Fig. 7 center). These peaks, not visible in raw feathers due to the complex native structure, become prominent as the treatment time is the longest (35 °C) or the temperature is the highest (75 °C). The longer hydrolysis time (270 min) at the lowest temperature (35 °C) was insufficient to cleave the disulfide bonds. Instead, this extended period and low temperature may have preserved the original S-S bonds in cystine from the feathers, making them more detectable in the Raman analysis. At 45 °C, the lowest S-S bond intensity could suggest that the moderate temperature, coupled with a relatively shorter time (180 min), was inadequate to make original S-S bonds more detectable, nor for significant disulfide bond cleavage and regeneration during acid precipitation. At 55 °C, even though the temperature is lower than 65 °C, there is a higher intensity of disulfide bonds, suggesting that the extended time (90 min) allows for a more thorough cleavage of the original disulfide bonds and a more complete regeneration during the acid precipitation stage at these temperatures. However, comparing 65 °C to 75 °C, the high difference in S-S intensities with a slight difference in hydrolysis time suggests that temperature plays a more crucial role than time in the disulfide bonds cleavage and their subsequent regeneration at these higher temperature ranges. The yields and S-S intensities at 45 °C and 65 °C are relatively higher, suggesting reduced keratin degradation under these conditions compared to 55 °C and 75 °C, respectively. In contrast, at 55 °C and 75 °C, the lower yields despite comparable S–S intensities indicate extensive disulfide bond cleavage and regeneration, but with greater keratin degradation into non-recoverable forms.

Stretching vibrations involving saturated and aromatic C-H bonds yield lower Raman intensities in keratin with increased temperature, compared with raw feathers and lower temperatures, indicating their unfolding and transformation. On the other hand, the in-plane vibrations of aromatic side chains—characteristic of amino acids like tyrosine (Tyr), tryptophan (Trp), and phenylalanine (Phe)—exhibit increased intensities at elevated temperatures, suggesting enhanced exposure of these residues or the formation of new chemical entities through oxidation or other reactions. Such changes could account for the observed darkening of the keratin colloid with rising temperatures.

**Fig. 10:**
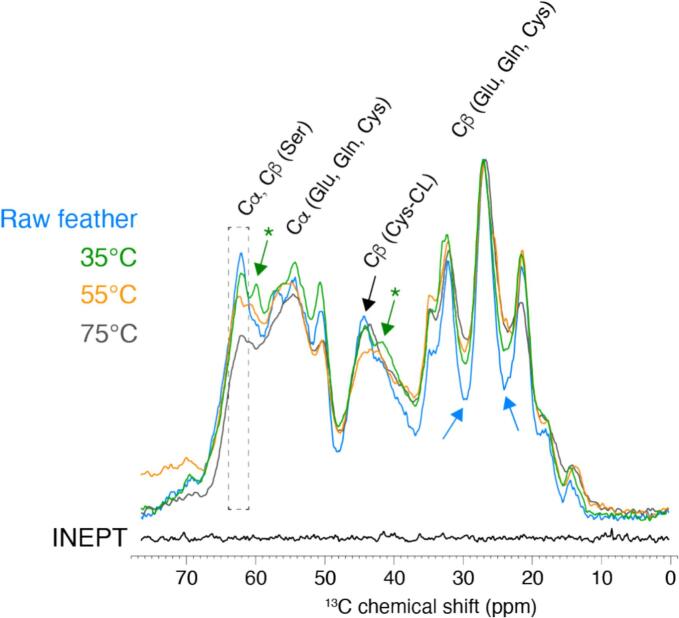
Solid-state NMR analysis of raw feather and recovered keratin from increasing hydrolysis temperature (35 °C, 55 °C, and 75 °C). 13C-detected cross-polarization and INEPT spectra were recorded at 500 MHz at a spinning frequency of 11 kHz, to probe rigid and mobile species of kerarin samples, respectively.

Solid-state NMR performed under magic-angle spinning conditions (MAS NMR) is a highly complementary tool to FTIR and Raman spectroscopies to derive information on the secondary structure content. In addition, MAS NMR can probe the local structural order of the keratin fibrous structure and complement macroscopic analysis by SEM. We employed ^13^C-detected cross-polarization (Fig. 10) to investigate the molecular conformation of hydrolyzed keratin at various temperatures (35 °C, 55 °C, and 75 °C) and compare it to an unprocessed raw feather sample. The MAS NMR spectrum of raw feathers shows a typical spectral pattern for the most abundant amino acids foiund in keratin (Glu, Gln, Cys and Ser), exemplified with the Serine Cβ contribution at ∼ 60–65 ppm (dashed box in Fig. 10). First, we used the observed ^13^C line-widths to decipher the degree of structural order and the presence of structural polymorphism in all samples. As exemplified by the foot of the peak at ∼ 27–28 ppm, we observed a more pronounced peak broadening for recovered keratin compared to raw feathers. This peak broadening suggests the presence of structural local disorder and polymorphism for the processed keratin, in line with the ultrastructural analysis performed by SEM. The presence of amorphous structure seems to be also correlated with the temperature, with wider broadening observed at 75 °C compared to 55 °C and 35 °C. We note that at a temperature of 35 °C, a noticeable level of rearrangement of the keratin organization is already observed.

Next, we investigated the region at ∼ 60–65 ppm to derive molecular changes at the level of the secondary structure. We observed an apparent decrease of the Serine Cβ contribution in α-helical conformation at ∼ 62–63 ppm with increased temperature, and we hypothesize a rearrangement of the local protein conformation in response to the temperature. The spectral pattern of recovered keratin samples at ∼ 57–60 ppm is quite diverse, and we observed the most noticeable change at a temperature of 35 °C with the presence of a strong contribution at ∼ 60 ppm (green arrow in Fig. 9). Although the complexity of such sample impeded an unambiguous assignment of this contribution, this chemical shift value encodes for a Cα position and reflect a substantial change of the local structure at 35 °C. Interestingly, the spectral patterns observed at higher temperatures (55 °C and 75 °C) are closer to raw feathers, suggesting the reformation of native structure at these high temperatures. To support this hypothesis, we exploited the spectral region at ∼ 40–45 ppm which encodes for cross-linked cysteine (Cβ contribution). We observed a decrease of the amount of cross-linked cysteine after the thermal treatments compared to native structures observed in raw feathers, with the most noticeable effect at 55 °C. A complex spectral pattern at ∼ 42 ppm is produced at 35 °C and 55 °C (green arrow in Fig. 9), suggesting that the local arrangement of cysteine residues is complex with the presence of disrupted non-covalent interactions and re-organized Cys-Cys interactions. Overall, it suggests a temperature-sensitive response of keratin fibrous structures to our process, with a structural disorder associated with the breaking of Cysteine disulfide bridges and further structural reorganization. Note that the sample processed at the highest temperature of 75 °C exhibited no clear signals in the ^13^C-detected INEPT (Fig. 9), suggesting that the thermal treatment does not significantly create minimal protein and peptide fragments, which would have been visible as mobile species in the INEPT experiment.

**Fig. 11:**
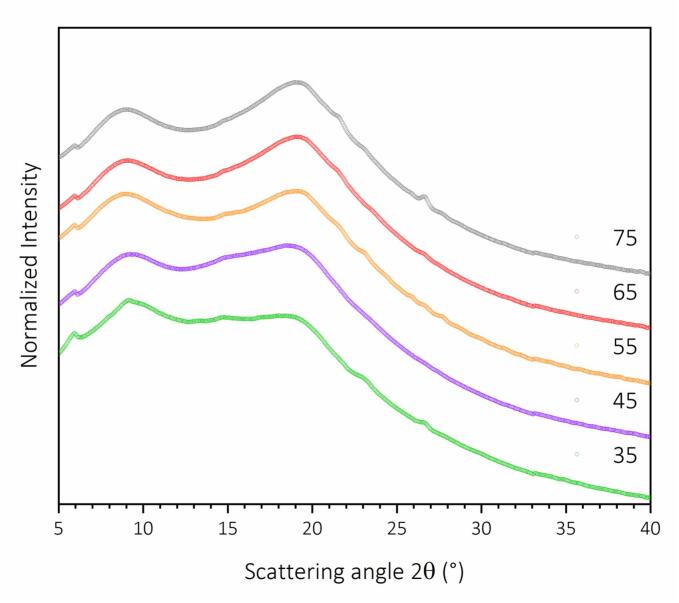
Comparison of X-ray diffraction (XRD) pattern for recovered keratin from increasing hydrolysis temperature (35 °C to 75 °C).

The XRD patterns shown in Fig. 11, measured on regenerated keratin obtained after ultrasound-assisted alkaline hydrolysis in a cup horn sonoreactor at different temperatures from 35 °C to 75 °C, confirm the results obtained by FTIR spectra, Raman spectroscopy, and solid-state NMR. All samples exhibit the semi-crystalline nature and characteristic broad diffraction halos typically associated with feather keratin, composed of partially ordered α-helix and β-sheet structures embedded in an amorphous matrix [19]. A shoulder around 2θ ≈ 9° is visible in all patterns and can be attributed to meridional reflections of α-helix structures, while the broad diffraction peak centered between 19° and 21° corresponds to equatorial reflections from β-sheet domains and random coil conformations [33].

The intensity of the α-helix-associated shoulder is slightly more defined in samples recovered at 35 °C, which could reflect a partial preservation of native helical structures during hydrolysis at lower temperatures. At higher hydrolysis temperatures (55 °C to 75 °C), the diffraction signal around 20° becomes more intense, suggesting a relative increase in disordered β-sheet and random coil regions, consistent with the unfolding and rearrangement of keratin chains. Overall, the diffraction patterns are broadly similar across the temperature range, with no clear emergence of sharp crystalline peaks, confirming that regenerated keratin remains predominantly amorphous. The absence of significant shifts in peak position implies that the secondary structure is largely preserved and that hydrolysis in the cup horn configuration, despite temperature changes, does not extensively alter the molecular packing of keratin.

### 4.3 Morphology analysis

**Fig. 12:**
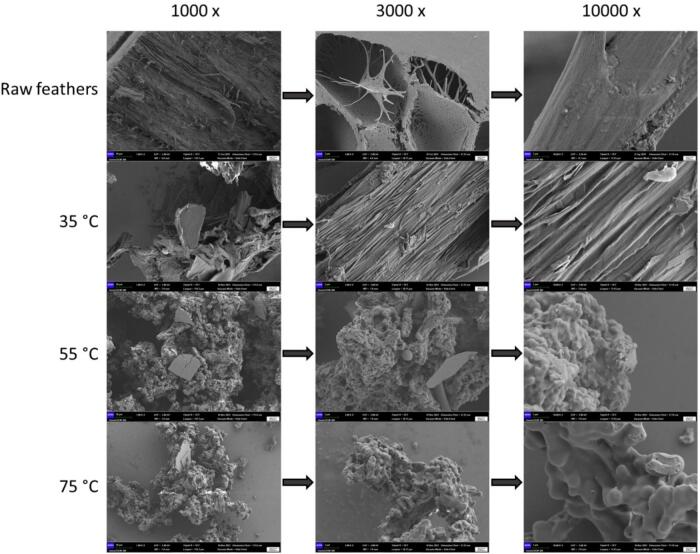
SEM images provide visual evidence of morphological changes from the fibrous structure at lower temperatures to more amorphous and compact aggregation structures at higher temperatures.

The SEM analysis at magnifications of 1,000×, 3,000×, and 10,000 × is presented in Fig. 12. It provides insights into the morphological changes in keratin at varying hydrolysis temperatures compared to raw feathers. Raw feathers exhibit a structured, fibrous arrangement consistent with original intact disulfide bridges conferring strength and resilience. At 35 °C, there is a slight disruption in the keratin structure, with a preservation of the fibrous morphology. This mild disruption may suggest that some disulfide bridges within the keratin remain unbroken, maintaining similarities to the unprocessed feather structure, especially regarding the native β-sheet (intermolecular antiparallel) organization, as observed in Table 3. At 55 °C, a visible shift from fibrous structure toward more amorphous and compact aggregation structures indicates further disulfide bond breakage and a possible transition towards reforming new structures. This is consistent with the amide I analysis showing a peak in β-sheet (inter AP)/side chains at this temperature, which could correspond to structural reorganization in line with disulfide bond regeneration. At 75 °C, the SEM images reveal a morphology similar to that of 55 °C, characterized by dense and irregular aggregations, aligning with amide I spectral findings of enhanced intra β-sheet and α-helix structures (Fig. 6). This suggests a thermally induced reconfiguration of keratin's secondary structure, where increased temperatures may facilitate the unfolding and subsequent refolding of protein chains, leading to a more pronounced exposure of previously internal structures or the formation of new stable configurations post-hydrolysis. The increase in compact aggregations observed at 55 °C and 75 °C can be attributed to the increased movement and interaction of hydrophobic regions due to thermal stress within keratin. These temperatures are sufficient to disrupt the more stable disulfide bonds and expose hydrophobic regions typically concealed within the protein structure. These regions, being averse to the aqueous environment, interact and coalesce, forming spherical aggregates. The globular structures are energetically favorable, minimizing the surface area exposed to water. The sheet-like structures identified in the SEM images at 55 °C and 75 °C could suggest the formation of sodium sulfides (see 1,000 × ). At elevated temperatures, the disulfide bonds in keratin are more likely to be broken, which could lead to the release of sulfur that reacts with sodium present in the solution to form sodium sulfides. The fact that these structures are not observed at lower temperatures supports the idea that they result from high-temperature reactions rather than sodium citrates, which would likely be present across all temperatures if they were a byproduct of the precipitation process with citric acid.

### 4.4 Thermal analysis

**Fig. 13:**
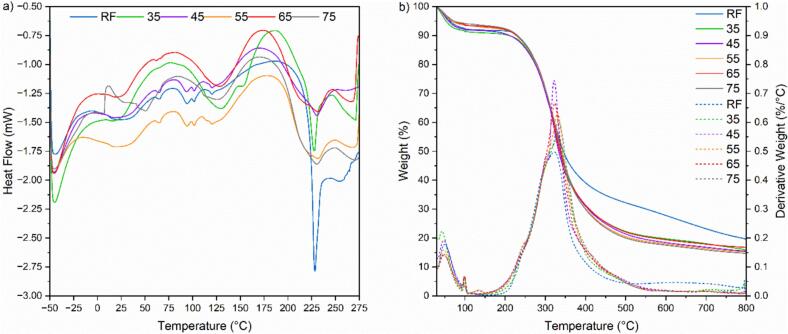
A) dsc thermograms and b)Thermogravimetric analysis (TGA) and derivative weight (DTG) curves of recovered keratin from increasing hydrolysis temperatures (35 °C to 75 °C), with raw feathers (RF) as a reference.

The DSC results in Fig. 13a show the endothermic peaks representing the heat capacity changes as proteins unfold. The first endothermic peaks observed between 100–150 °C across all samples indicate water loss and the unfolding of protein structures, primarily through the disruption of hydrogen bonds. The broader transitions in the 35 °C, 65 °C, and 75 °C samples could signify a hydrogen bonding network within the keratin, which aligns with the amide I analysis in the ATR-IR findings that suggest higher α-helix structures at these temperatures. The similarity of the 45 °C and 55 °C samples to raw feathers might be explained by the less α-helix structures reconstitution at these temperatures. In the 200–250 °C range, where α-helix denaturation is observed, the 35 °C sample exhibits a sharper endothermic response superposing with that of raw feather. This may be due to the preservation of secondary structures that remain intact. Conversely, the higher temperature samples show delayed and less intense peaks, suggesting the formation of more amorphous structures and possibly new, more thermally resistant α-helix structures. Finally, the 250–280 °C range reflects the β-sheet and disulfide bond decomposition. The more pronounced decomposition in the 35 °C sample could indicate a greater quantity of regenerated disulfide bonds, providing additional thermal stability and potentially explaining the increased resistance to denaturation observed in the 200–250 °C range compared to raw feathers. The minimal response observed in this range at 45 °C, where hydrolysis duration was shorter (180 min compared to 270 min at 35 °C), suggests that longer exposure has partially led to the breakdown of disulfide bonds and their subsequent reformation during acid precipitation while retaining the original S-S bonds of the feathers. This trend was followed by temperatures 75 °C, 55 °C, and 65 °C, as shown in Raman analysis.

The thermogravimetric analysis (TGA) of raw feather and keratin processed at varying temperatures Fig. 13b, provides insight into the material's thermal stability and decomposition characteristics. Initial weight loss for all samples below 250 °C, apparent at around 100 °C, is attributed to the evaporation of free water. A subsequent plateau up to 150 °C likely indicates the release of bound water, suggesting the breakdown of weak hydrogen bonds in keratin. A distinct slope change in the derivative thermogravimetry (DTG) profiles between 230 – 250 °C in feathers corresponds to the thermal disruption of α-helix structures. Beyond 250 °C, the raw feathers exhibit the slowest degradation rate, demonstrating the inherent thermal resistance of the native disulfide bonds. This is followed by keratin processed at temperatures 35 °C, 75 °C, and 55 °C, respectively, with a progressive shift of the DTG peaks at these temperatures. This shift suggests a sequence of increasing thermal stability, potentially due to the formation of regenerated disulfide bonds, as observed in Raman spectroscopy and the DSC analysis.

On the other hand, keratins processed at 45 °C and 65 °C demonstrate a faster degradation rate, with DTG peaks appearing at lower temperatures. This behavior can be interpreted as a consequence of reduced thermal stability owing to the less complex structure than the raw feather and further influenced by the lack of regenerated disulfide bonds. The char residue analysis reveals that the highest char yield is observed in raw feathers (19.41 %), indicative of a robust, thermally resistant structure. Keratin from lower temperature treatments also shows a higher char yield (15.78 %) than that processed at elevated temperatures (14.69 % at 75 °C). This result could indicate the preservation of native structures that resist thermal degradation.

## 5 Conclusion

This study presents an innovative approach for the valorization of duck feather waste through ultrasound-assisted alkaline hydrolysis in a Cup Horn sonoreactor. This configuration provided homogeneous acoustic energy distribution and controlled cavitation, enabling precise modulation of keratin depolymerization and structural reorganization under mild conditions. The process revealed three distinct regimes governed by temperature: at 35 °C, cystine preservation and α-helix retention dominated; at 55 °C, partial disulfide bond rupture and regeneration occurred, accompanied by visible color changes due to chromophore exposure; and at 75 °C, extensive β-sheet formation and uniform molecular reorganization were achieved. Spectroscopic (ATR-IR, Raman, solid-state ^13^C NMR), electrophoretic, and morphological (SEM, XRD) analyses consistently demonstrated that temperature modulates the balance between disulfide cleavage, chain unfolding, and refolding into ordered domains. Thermal analysis confirmed enhanced stability in keratins recovered at higher temperatures, associated with regenerated disulfide bridges. Overall, this work provides mechanistic insights into the interplay between sonication, temperature, and chemical integrity in keratin recovery. Although this study did not directly quantify cystine and lanthionine, future research will implement dedicated HPLC-based amino acid analysis to complement the structural characterization and confirm the extent of cystine preservation in comparison to lanthionine formation under different hydrolysis conditions. The Cup Horn sonoreactor proves to be a powerful platform for tuning keratin properties through controlled ultrasound–alkaline interaction. The proposed strategy represents a scalable, energy-efficient, and environmentally sound route for transforming feather waste into value-added biopolymers with tailored molecular and structural features.

## CRediT authorship contribution statement

**Nidal Del Valle Raydan:** Writing – original draft, Visualization, Methodology, Investigation, Formal analysis, Data curation, Conceptualization. **Antoine Loquet:** Writing – review & editing, Supervision, Investigation. **Birgit Habenstein:** Writing – original draft, Investigation, Formal analysis, Data curation. **Brice Kauffmann:** Writing – original draft, Investigation, Formal analysis, Data curation. **Gregory Chatel:** Writing – review & editing, Visualization, Supervision, Conceptualization. **Eduardo Robles:** Writing – review & editing, Supervision, Project administration, Conceptualization.

## Declaration of competing interest

The authors declare that they have no known competing financial interests or personal relationships that could have appeared to influence the work reported in this paper.
